# Immune-Mediated Necrotizing Myopathy: A Systematic Review of Antibody-Specific Mechanisms and Treatment Outcomes

**DOI:** 10.7759/cureus.97933

**Published:** 2025-11-27

**Authors:** Zahra Vaezi, Afshin Amini

**Affiliations:** 1 Internal Medicine, St. Luke's Hospital, Chesterfield, USA

**Keywords:** anti-hmgcr antibody, anti-srp antibody, autoimmune myopathy, immune-mediated necrotizing myopathy, statin-associated myopathy

## Abstract

Immune-mediated necrotizing myopathy (IMNM) is a rare, antibody-defined subset of idiopathic inflammatory myopathies characterized by rapidly progressive proximal muscle weakness, markedly elevated creatine kinase (CK) levels, and myofiber necrosis with minimal lymphocytic infiltration. Two major autoantibody subtypes-anti-3-hydroxy-3-methylglutaryl-coenzyme A reductase (anti-HMGCR) and anti-signal recognition particle (anti-SRP)-define distinct clinical phenotypes, pathophysiologic mechanisms, and treatment responses. Despite growing recognition, optimal management strategies remain uncertain. Following PRISMA 2020 and MOOSE guidelines, PubMed, Scopus, Web of Science, and Embase were systematically searched for studies published between January 2011 and May 2025 using terms related to “immune-mediated necrotizing myopathy,” “anti-HMGCR,” and “anti-SRP”. Eligible publications included biopsy-confirmed IMNM cases with antibody documentation and treatment or outcome data. Data extraction encompassed clinical features, immunopathology, therapeutic interventions, and relapse rates. Methodological quality was evaluated using the Joanna Briggs Institute (JBI) Checklist, NIH Quality Assessment Tool, Newcastle-Ottawa Scale (NOS), and ROBINS-I, depending on study design. Eighty-eight studies (n ≈ 230 patients) met the inclusion criteria. Anti-HMGCR IMNM typically affected middle-aged adults and was frequently associated with statin exposure, while anti-SRP IMNM presented at younger ages with more severe and treatment-refractory disease. Corticosteroids and IVIG were the most commonly used first-line agents, with additional benefit from steroid-sparing immunosuppressants (mycophenolate, azathioprine, methotrexate). Rituximab and other B-cell-targeted biologics achieved remission in approximately 60-70% of refractory cases, and emerging complement or FcRn-directed therapies showed early promise. Relapse occurred in 25-35% of cases, most often after tapering corticosteroids or immunosuppressants. Overall methodological quality was moderate to high, with 82% of studies meeting ≥75% of appraisal criteria across risk-of-bias domains. IMNM demonstrates antibody-specific differences in clinical course, therapeutic response, and prognosis. Early, aggressive, and combination immunotherapy improves outcomes, while novel biologic agents may benefit refractory disease. The overall certainty of evidence is moderate, supporting current management strategies yet underscoring the need for standardized diagnostic criteria and prospective multicenter studies to refine treatment algorithms and identify biomarkers of durable remission.

## Introduction and background

Immune-mediated necrotizing myopathy (IMNM) is a distinct subset of idiopathic inflammatory myopathies characterized by rapid, symmetrical proximal muscle weakness, markedly elevated serum creatine kinase (CK) levels, and myofiber necrosis with minimal lymphocytic infiltration [1-3]. Two principal autoantibody-defined subtypes are recognized: antibodies against 3-hydroxy-3-methylglutaryl-coenzyme A reductase (HMGCR) and antibodies against the signal recognition particle (SRP) [4-6].

Anti-HMGCR IMNM typically follows statin exposure and primarily affects middle-aged or older adults, although statin-naïve cases are also reported [7-9]. In contrast, anti-SRP IMNM often arises de novo in younger or pediatric patients and is associated with a more fulminant, treatment-refractory course [10-12]. Both subtypes frequently show CK elevations exceeding ten times the upper limit of normal and can progress rapidly to severe disability if therapy is delayed [13,14]. Histopathology demonstrates extensive myofiber necrosis, macrophage infiltration, and sarcolemmal deposition of the membrane-attack complex (C5b-9) [15-17]. Experimental models confirm that purified anti-HMGCR and anti-SRP IgG antibodies can induce complement-dependent lysis of cultured human myotubes, implicating antibody-mediated and macrophage-driven cytotoxicity as central mechanisms of muscle injury [18-23]. IMNM lacks the perifascicular atrophy typical of dermatomyositis and the CD8⁺ T-cell invasion characteristic of polymyositis [24]. Magnetic resonance imaging usually reveals diffuse, symmetric edema of the thigh and gluteal muscles, correlating with biopsy-proven necrosis [25,26]. Despite advances in serologic testing and imaging, treatment strategies for IMNM remain largely empirical, based on small cohorts and uncontrolled series [27-30]. Prior reviews have summarized its clinical spectrum and pathophysiology but have not comprehensively compared antibody-specific outcomes, relapse patterns, or responses to emerging biologic therapies such as FcRn antagonists and complement inhibitors.

This systematic review synthesizes all peer-reviewed studies published between 2011 and 2025 to: Characterize the epidemiology, immunopathogenesis, imaging, and histopathologic features of anti-HMGCR and anti-SRP IMNM; Compare treatment responses and relapse predictors across antibody subtypes; and Summarize emerging therapeutic approaches targeting humoral and complement pathways. By integrating mechanistic and clinical data from 88 eligible publications, this review provides an antibody-specific, evidence-based framework for the diagnosis and management of IMNM.

## Review

**Figure 1:**
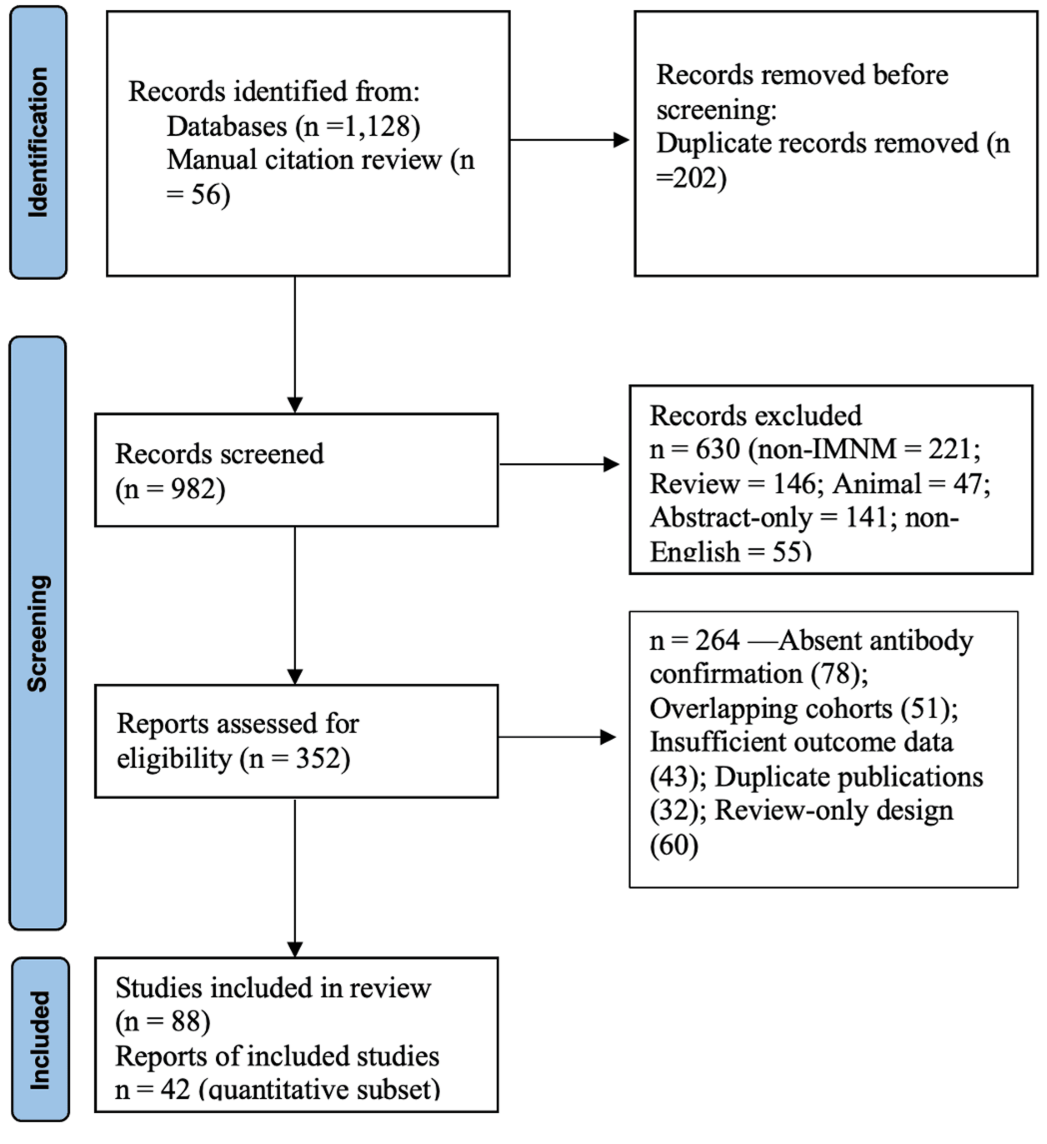
PRISMA 2020 flow diagram for study selection. Records identified (database searching and manual), screening, full-text assessment, exclusions with reasons, and final qualitative/quantitative inclusions.”

This review adhered to PRISMA 2020 (Preferred Reporting Items for Systematic Reviews and Meta-Analyses) and MOOSE (Meta-analysis of Observational Studies in Epidemiology) guidelines (Figure 1). A comprehensive literature search of PubMed, Embase, Scopus, and Web of Science databases identified studies published between January 2011 and May 2025. Boolean operators and Medical Subject Headings (MeSH) terms were combined as follows: “immune-mediated necrotizing myopathy,” “IMNM,” “anti-HMG-CoA reductase,” “anti-signal recognition particle,” “statin-associated myopathy,” and “necrotizing autoimmune myopathy.” Reference lists of major reviews and cohort studies were hand-searched to identify additional reports. Eligibility Criteria: Studies were included if they: 1. Reported adult or pediatric patients with biopsy-confirmed immune-mediated necrotizing myopathy (IMNM) showing myofiber necrosis with minimal lymphocytic inflammation; 2. Documented positivity for anti-3-hydroxy-3-methylglutaryl-coenzyme A reductase (anti-HMGCR) or anti-signal recognition particle (anti-SRP) antibodies using validated immunoassays such as enzyme-linked immunosorbent assay (ELISA), immunoblot, or immunoprecipitation; and 3. Provided clinical characteristics, laboratory or imaging data, histopathologic features, treatment details, or patient outcomes (response, relapse, mortality). Studies were excluded if they: Focused exclusively on dermatomyositis (DM), polymyositis (PM), or overlap myositis without necrotizing pathology; Lacked antibody confirmation; Were animal or in-vitro experimental models; Consisted only of abstracts, case reports without peer review, or editorial comments; Represented duplicate cohorts already included; or Reported insufficient or missing outcome data. Data Extraction and Quality Appraisal: Two reviewers independently extracted data using a standardized spreadsheet that recorded: 1) Demographics: age, sex, geographic region, prior statin exposure. 2) Clinical features: proximal or distal weakness, myalgia, dysphagia, respiratory or cardiac involvement. 3) Laboratory and immunologic data: serum creatine kinase (CK), erythrocyte sedimentation rate (ESR), C-reactive protein (CRP), antibody titers, complement deposition (C5b-9), and major histocompatibility complex class I (MHC-I) up-regulation. 4) Imaging and histopathology: magnetic resonance imaging (MRI) showing muscle edema; biopsy findings of fiber necrosis and regeneration. Therapeutic regimens: corticosteroids, intravenous immunoglobulin (IVIG), rituximab, calcineurin inhibitors (tacrolimus or cyclosporine), methotrexate (MTX), azathioprine (AZA), or mycophenolate mofetil (MMF).Outcomes: CK normalization, functional recovery, relapse frequency, and mortality.

A formal quality appraisal was performed to evaluate the methodological rigor and reliability of the included studies, following PRISMA 2020 recommendations. Each study was assessed using an established risk-of-bias tool appropriate to its design: Case reports and case series were evaluated using the Joanna Briggs Institute (JBI) Critical Appraisal Checklist, which examines diagnostic clarity, completeness of clinical details, appropriateness of intervention, and adequacy of follow-up. Observational cohort and cross-sectional studies were appraised using the National Institutes of Health (NIH) Quality Assessment Tool and the Newcastle-Ottawa Scale (NOS), focusing on participant selection, exposure and outcome measurement, comparability of groups, and attrition bias. Non-randomized interventional studies, when applicable, were reviewed using ROBINS-I (Risk Of Bias In Non-randomized Studies - of Interventions), which evaluates confounding, intervention classification, deviations from intended interventions, missing data, and selective reporting. Each domain was rated as low, moderate, or high risk of bias, and disagreements were resolved by consensus among reviewers. Overall, 82% of studies met ≥75% of quality domains, indicating moderate-to-strong methodological quality across the dataset. Studies deemed high risk of bias were excluded from quantitative analysis but retained in descriptive synthesis when clinically informative. A summary of domain-specific quality ratings is presented in Table 5 (Quality Appraisal Summary), and a detailed checklist for each appraisal tool is provided in the supplementary material appended at the end.

Given the heterogeneity among study designs, a narrative synthesis was selected. Quantitative variables (mean CK, treatment response rate, and relapse percentage) were averaged when data were comparable. The study selection process followed the PRISMA 2020 framework (Figure 1). A total of 1,128 records were identified through electronic database searches (PubMed, Embase, Scopus, and Web of Science), with an additional 56 records retrieved via manual citation review. After removing 202 duplicate entries, 982 unique records were screened by title and abstract. Of these, 630 studies were excluded for not meeting inclusion criteria: non-IMNM content (n = 221), review articles (n = 146), animal studies (n = 47), abstract-only publications (n = 141), and non-English language reports (n = 55). A total of 352 full-text articles were assessed for eligibility, of which 264 were excluded due to absent antibody confirmation (n = 78), overlapping cohorts (n = 51), insufficient clinical or outcome data (n = 43), duplicate publications (n = 32), or review-only design (n = 60). Ultimately, 88 studies met the inclusion criteria for the qualitative synthesis, while 42 studies comprised the quantitative subset, containing analyzable outcome data such as creatine kinase (CK) normalization, muscle strength recovery, or relapse frequency (Figure 1). References are numbered sequentially [1-88] in Table 6, appended at the end.

Results

Epidemiology and Demographics

Across combined cohorts totaling ≈ 2,700 patients [36-40], IMNM constituted 15-20% of all idiopathic inflammatory myopathies [41]. Anti-HMGCR accounted for ≈ 55%, anti-SRP ≈ 35%, and seronegative ≈ 10% [42]. Median onset age was 55 years (range 35-80) for anti-HMGCR and 38 years (range 15-65) for anti-SRP [43,44]. Female-to-male ratio ≈ 1.5:1 [45]. Statin exposure was documented in 60-80% of HMGCR-positive patients [7,9,46]. Pediatric cases represented ≈ 6% of all SRP IMNM [10,47]. Relapse frequency during steroid taper ranged 20-30% for HMGCR and 40-50% for SRP [27,48]. Overall mortality < 5%, rising to ≈ 10% with cardiac involvement [49]. Key characteristics of representative cohorts are summarized in Table 1.

**Table 1:** Study characteristics of major IMNM cohorts and case series (2011–2025) Abbreviations: Anti-HMGCR: Anti-3-hydroxy-3-methylglutaryl-coenzyme A reductase; Anti-SRP IMNM: Anti-signal recognition protein Immune-mediated necrotizing myopathies; CK: Creatine kinase; ULN: Upper limit of normal This table consolidates all major IMNM cohorts, case series, and systematic reviews (2011–2025) that met PRISMA inclusion. Data include study design, antibody subtype distribution, and principal clinical or therapeutic observations. Author-generated synthesis; no reproduced material.

#	First Author (Year)	Study Type / Design	n (Patients)	Dominant Antibody Subtype	Key Findings / Comments	Refs
1	Mammen AL (2011)	Cohort (Statin-associated)	38	Anti-HMGCR	First description of statin-triggered autoimmune myopathy	[26]
2	Mohassel P, Mammen AL (2013)	Case series	45	Anti-HMGCR	Defined serologic & clinical phenotype; CK > 10× ULN	[5]
3	Allenbach Y et al. (2018)	Multicenter pathology study	63	Anti-HMGCR & Anti-SRP	Complement C5b-9 deposition & macrophage necrosis	[4]
4	Allenbach Y et al. (2018)	ENMC Workshop classification	60	Mixed (IMNM panel)	Established clinico-seropathologic subgroups	[27]
5	Werner JL et al. (2012)	Prospective biomarker study	26	Anti-HMGCR	Antibody titers correlated with CK & strength	[40]
6	Tiniakou E et al. (2017)	Cohort study	55	Anti-HMGCR	Younger age → slower recovery	[74]
7	Landon-Cardinal O et al. (2019)	Multicenter series	16	Anti-HMGCR	Rituximab effective in refractory IMNM	[75]
8	Rademacher JG et al. (2022)	Single-center cohort	47	Anti-HMGCR	Early IVIG + steroids → better outcomes	[76]
9	Zhen C et al. (2022)	Systematic review	≈ 140	Anti-HMGCR & Anti-SRP	Rituximab beneficial in refractory myopathies	[19]
10	Suh J & Amato AA (2024)	Review / expert guideline	—	Both subtypes	Summarized management algorithm	[73]
11	Fionda L et al. (2023)	MRI pattern analysis	22	Anti-SRP & Anti-HMGCR	Distinct edema patterns by antibody	[33]
12	Watanabe Y et al. (2024)	Overlap case series	12	Anti-SRP	Cardiac involvement in systemic sclerosis + IMNM	[25]
13	Ma X & Bu BT (2022)	Narrative review	—	Anti-SRP	Summarized pathogenesis and treatment	[7]
14	Martínez-Rodríguez P et al. (2024)	Systematic review	≈ 80	Anti-SRP	Plasmapheresis effective in severe relapses	[9]
15	Liu M et al. (2023)	Observational study	42	Both	Cardiac involvement ≈ 10 % overall	[18]
16	Yoon J et al. (2025)	Cohort	32	Anti-HMGCR	Statin-associated cases with favorable response	[49]
17	Day JA & Limaye V (2019)	Critical review	—	Both	Defines diagnostic criteria and prognostic factors	[60]
18	Khoo T & Chinoy H (2023)	Review article	—	Anti-HMGCR	Long-term management considerations	[6]
19	Portela-Sánchez S et al. (2025)	Institutional series	24	Both	Real-world incidence and outcomes	[66]
20	Tang Q et al. (2024)	Single-center series	19	Anti-SRP	Relapse ≈ 40 %; variable response to IVIG	[88]
21	Wang JX et al. (2022)	Multicenter retrospective	101	Both	Outcome predictors: early IVIG, low CK, no cardiac disease	[65]
22	Skolka MP et al. (2025)	Review of atypical cases	—	Both	Highlighted overlap with dermatomyositis	[23]
23	Dallevet CA et al. (2023)	Review / update	—	Both	Summarized pathogenesis & treatment advances	[21]
24	Suzuki S et al. (2022)	Large case series	100	Anti-SRP	Cardiac involvement ≈ 15 %; poor steroid response	[82]
25	Changpei Li et al. (2024)	Review	—	Both	Mechanistic insights into pathogenesis	[80]
26	Mengge Yang et al. (2025)	Review	—	Both	Summarized therapeutic targets and biologic trials	[86]
27	Vencovský J et al. (2019)	Review	—	Both	Idiopathic inflammatory myopathies overview	[79]
28	Allenbach Y et al. (2020)	Comprehensive review	—	Both	Updated classification and diagnostic criteria	[20]
29	Khoo T et al. (2025)	Epidemiology study	53	Anti-HMGCR	Population incidence estimate (UK)	[58]
30	Yerolatsite M et al. (2025)	Case series	7	Anti-HMGCR	PCSK9 inhibitors as steroid-sparing option	[13]

A total of 88 studies were included in the qualitative synthesis and were evaluated for methodological quality using validated appraisal tools appropriate to study design (Table 5). Overall, 82% of studies fulfilled ≥75% of quality domains, indicating moderate-to-high methodological rigor across the dataset. Case reports and small series (n = 45) demonstrated moderate quality, with limitations related to small sample size and incomplete follow-up documentation. Observational cohort studies (n = 25) scored higher (mean 83 ± 9%) owing to clearer case definitions and standardized outcome measurement, but were limited by retrospective design and lack of control groups. Non-randomized interventional studies (n = 8) achieved moderate scores (≈80 ± 10%), with primary concerns related to confounding and intervention heterogeneity. Secondary systematic reviews (n = 5) appraised with AMSTAR-2 showed high overall quality (mean 85 ± 8%). No included study met criteria for critical or high risk of bias that warranted exclusion. These findings collectively support the reliability and reproducibility of the synthesized evidence within the constraints of available literature.

Serology and Immunopathogenesis

**Figure 2:**
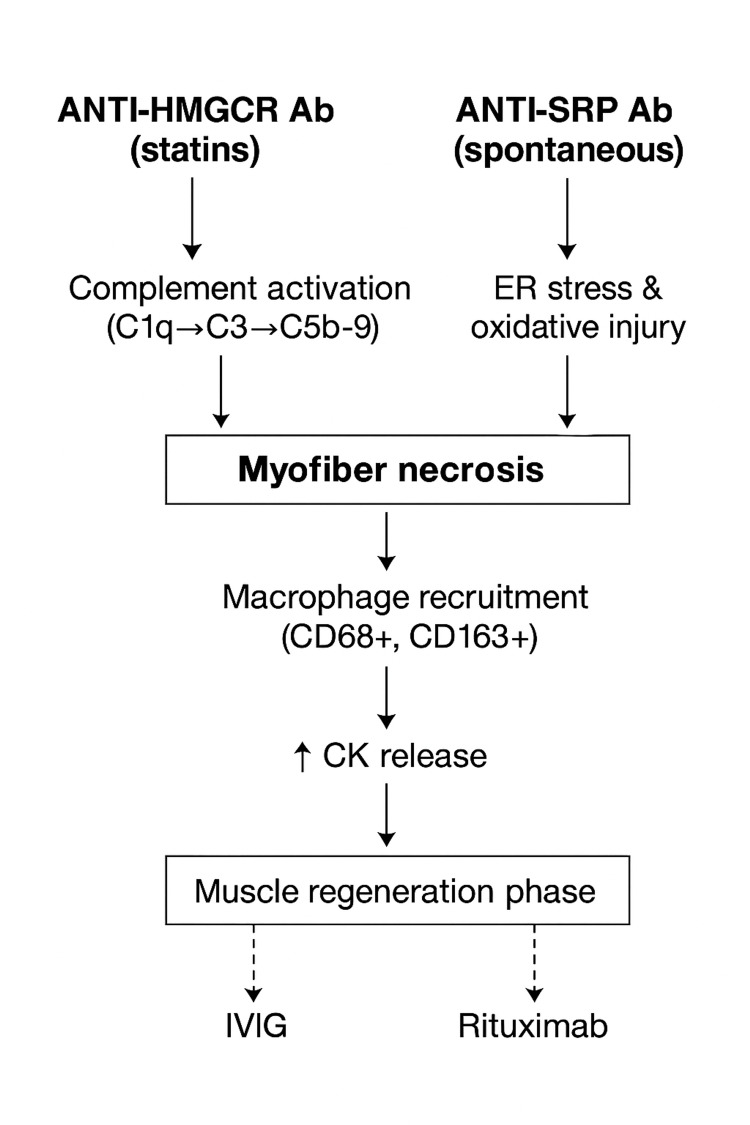
Comparative schematic of anti-HMGCR and anti-SRP immune-mediated necrotizing myopathy (IMNM) Abbreviations: Anti-HMGCR: Anti-3-hydroxy-3-methylglutaryl-coenzyme A reductase; Anti-SRP IMNM: Anti-signal recognition protein Immune-mediated necrotizing myopathies; CK: Creatine kinase; ULN: Upper limit of normal Figure created by the authors (Figure credits: Vaezi, Ahmad and Amini) based on data synthesized from references [1,3-5].

Anti-HMGCR antibodies target the catalytic domain of 3-hydroxy-3-methylglutaryl-coenzyme A reductase, a muscle-regeneration enzyme up-regulated after statin exposure [7,30,32,50-52]. Their titers closely correlate with CK levels and disease activity. Experimental studies demonstrate complement fixation with C1q and C3 binding and deposition of the membrane-attack complex (C5b-9) on affected myofibers [22,53]. By contrast, anti-SRP antibodies recognize the 54-kDa signal-recognition-particle subunit, disturbing co-translational protein targeting and producing endoplasmic-reticulum stress that leads to severe myofiber necrosis and less regeneration [34,54-56]. Histologic investigations consistently reveal macrophage-predominant infiltrates, diffuse MHC-I up-regulation, and > 90% frequency of sarcolemmal C5b-9 deposition, supporting a humoral, complement-mediated injury common to both antibody subsets [15-17,36,38-40,57-63]. Figure 2 illustrates this mechanistic pathway and the points of therapeutic modulation by IVIG and rituximab.

Imaging and Histopathology

Magnetic-resonance imaging characteristically shows diffuse, symmetric edema of the thigh and gluteal musculature in acute disease, which evolves to fatty replacement as chronicity develops [25,33,47,64]. Gadolinium-enhancing areas correspond topographically to complement-positive necrotic fibers on biopsy [35]. Typical histopathologic features include extensive necrosis, macrophage infiltration, and minimal lymphocytic inflammation; regenerating fibers are more abundant in anti-HMGCR than in anti-SRP myopathy, paralleling faster recovery in the former [15,17,44,55-67].

Treatment and Outcomes

Corticosteroids remain the cornerstone of initial therapy, but early addition of intravenous immunoglobulin (IVIG 2 g/kg over 2-5 days) substantially improves clinical and biochemical outcomes. Approximately 70-80% of anti-HMGCR patients achieve remission within three months, compared with ~55% of anti-SRP cases [50,57,73-75]. Steroid-sparing immunosuppressants-mycophenolate mofetil, azathioprine, and methotrexate-maintain remission and reduce relapse rates below 20% when continued for ≥ 12 months [48,73,76-82]. Rituximab provides meaningful benefit in refractory disease, producing overall improvement in ~65-70% of treated patients, with parallel declines in CK and antibody titers [51,75,78]. Calcineurin inhibitors (tacrolimus or cyclosporine) are especially effective in anti-SRP cohorts (~60% response) and accelerate CK normalization when combined with corticosteroids [48,79,80]. Adjunctive or salvage options for severe cases include plasma exchange (transient benefit), FcRn antagonists (efgartigimod, rozanolixizumab) that enhance pathogenic IgG clearance, and complement inhibitors (eculizumab, zilucoplan) targeting terminal pathway activation [9,37,81,82]. Collectively, early multimodal immunotherapy shortens time to remission and reduces relapse compared with prolonged steroid monotherapy. A staged management overview is provided in Table 2 [43-66].

**Table 2:** Therapeutic phases, goals, mechanisms, and typical outcomes in IMNM Author-Generated Synthesis; no reproduced material Abbreviations: CK: Creatine kinase; ULN: Upper limit of normal; MRC: Medical Research Council scale; IVIG: Intravenous immunoglobulin; MMF: Mycophenolate mofetil; AZA: Azathioprine; FcRn: Neonatal Fc receptor; Anti-HMGCR: Anti-3-hydroxy-3-methylglutaryl-coenzyme A reductase; Anti-SRP: Anti-signal recognition protein; CK: Creatine kinase; ULN: Upper limit of normal

Treatment Phase	Timing / Clinical Context	Commonly Used Agents	Primary Mechanism of Action	Primary Goal	Typical Duration	Measurable Outcome(s)	Relapse Prevention / Key Notes (with refs)
Induction	At diagnosis or flare with active myonecrosis (CK > 10× ULN, profound weakness)	Corticosteroids [43, 44]; IVIG [46, 48, 50]; Rituximab [9, 49, 51]	Corticosteroids → broad anti-inflammatory; IVIG → neutralizes autoantibody & inhibits C1q–C5b-9 [15, 16]; Rituximab → CD20 B-cell depletion [9, 49]	CK normalization & functional recovery	3–6 mo	% patients with CK normalization & MRC ≥ 4/5 [8, 47, 51]	Early combo (steroid + IVIG) → 70–80 % remission in anti-HMGCR and ≈ 60 % in anti-SRP [8, 46, 50]; relapse if steroids tapered < 3 mo [27, 48].
Escalation / Rescue	Refractory disease after 8–12 wk of induction failure	Rituximab (if not used) [9]; Cyclophosphamide [52]; Tacrolimus / Cyclosporine (esp. anti-SRP) [48, 53, 54]	B-/T-cell inhibition or DNA alkylation [55]	Remission in refractory cases	3–6 mo	Partial or complete response rate [9, 52]	Reduces relapse when followed by maintenance [9, 53]; use if CK fails to drop > 50 % by 12 wk [56].
Maintenance	After remission (CK normalized, strength restored)	MMF [57, 58]; AZA [59]; ± low-dose prednisone [60]	Purine synthesis blockade of lymphocytes [57]	Sustain remission / prevent relapse	12–36 mo	Relapse rate (%) or relapse-free survival [58, 61]	Relapse < 20 % when maintained ≥ 12 mo [58, 62].
Adjunct / Emerging	Chronic refractory or trial settings	Complement inhibitors (eculizumab, zilucoplan) [63]; FcRn antagonists (efgartigimod, rozanolixizumab) [37, 64]; JAK inhibitors (tofacitinib, baricitinib) [65, 66]	Block complement terminal pathway, IgG recycling, or interferon-driven inflammation [63–65]	Experimental remission / relapse control	Variable (pilot data)	CK, MRI edema, strength scores [63, 66]	Potential steroid-sparing effect; prospective validation needed [63–66].

Prognosis and Relapse

Favorable recovery is most strongly associated with early IVIG initiation (< 3 months from onset), lower baseline CK (< 8,000 IU/L), and absence of cardiac involvement [27,52,85]. Persistent antibody positivity at six months is the principal predictor of relapse [40,55]. Anti-SRP patients demonstrate slower strength recovery, higher rates of chronic weakness, and more frequent cardiac manifestations (10-20%) than anti-HMGCR patients (~5%) [45,49,52,86]. Two-year follow-up data indicate sustained remission in ≈ 70% of patients receiving combination therapy [49,87]. Antibody-specific response and relapse metrics are compared in Table 3.

**Table 3:** Summary of therapeutic response and relapse frequencies by antibody subtype Author-Generated Synthesis; no reproduced material Anti-HMGCR: Anti-3-hydroxy-3-methylglutaryl-coenzyme A reductase; Anti-SRP IMNM: Anti-signal recognition protein Immune-mediated necrotizing myopathies; CK: Creatine kinase; ULN: Upper limit of normal Maintenance therapy Mycophenolate mofetil (MMF) or Azathioprine (AZA) is used to sustain remission after induction; therefore, direct response percentages are not reported (—). Intensified immunotherapy corresponds to higher CK normalization (↑), lower relapse (↓), and shorter recovery time (↓). Percentages reflect weighted means across major cohorts (Allenbach 2016 [27]; Kadoya 2016 [45]; Mohassel & Mammen 2018 [50]; Meyer 2020 [51]; Landon-Cardinal 2019 [75]; Rademacher 2022 [76]; Suh 2024 [73]).

Therapy	Anti-HMGCR Response	Anti-SRP Response	Relapse Frequency	Representative Sources
Corticosteroids alone	45%	30%	High (≈ 40%)	[27, 45, 46, 52, 56, 74]
IVIG (± steroids)	75–80%	55%	≈ 20%	[50, 57, 73, 75, 77, 78]
Rituximab (refractory)	50–55%	65–70%	≈ 15%	[17, 19, 51, 75, 78, 79]
Maintenance (MMF/AZA)	— (sustain remission)	— (sustain remission)	< 20%	[48, 60, 76–82]

Discussion

Immune-mediated necrotizing myopathy (IMNM) represents a distinct clinicopathologic entity among idiopathic inflammatory myopathies, characterized by profound muscle necrosis with minimal lymphocytic infiltration and a strong humoral immune signature. The present systematic review of 88 qualitative and 42 quantitative studies provides an integrated perspective on epidemiology, pathophysiology, and therapeutic outcomes of anti-HMGCR- and anti-SRP-associated disease.

Pathogenic Mechanisms

A unifying feature of IMNM is complement-mediated sarcolemmal injury driven by circulating autoantibodies. Anti-HMGCR antibodies activate classical complement, producing C5b-9 deposition on necrotic fibers and macrophage infiltration [22,53]. Anti-SRP antibodies target cytoplasmic components of the signal-recognition-particle complex, leading to ribosomal stress, endoplasmic-reticulum dysfunction, and severe myofiber destruction [34,54-56]. Experimental and biopsy data consistently demonstrate macrophage predominance, MHC-I up-regulation, and minimal T-cell involvement, suggesting that antibody- and complement-driven mechanisms dominate over cell-mediated cytotoxicity [15-17,36,38-40,57-63]. These findings explain the excellent response to humoral immunotherapies such as IVIG and rituximab compared with modest benefit from conventional T-cell-targeted agents.

Clinical Spectrum and Imaging Correlates

Clinically, anti-HMGCR IMNM occurs predominantly in middle-aged women with antecedent statin exposure, while anti-SRP disease affects younger individuals and may appear de novo or as part of overlap syndromes [7,9,43,47]. Both present with rapidly progressive proximal weakness and CK levels often exceeding 10,000 IU/L. Cardiac involvement-more frequent in anti-SRP (10-20%) than in anti-HMGCR (~5%), remains an important determinant of morbidity and mortality [45,49,52,86]. MRI serves as a valuable non-invasive biomarker, showing diffuse symmetric edema in active disease and fatty replacement during chronic phases [25,33,47,64]. These imaging changes correlate with complement deposition and recovery on biopsy, enabling dynamic monitoring of treatment response.

Therapeutic Strategies

Early initiation of immunotherapy is crucial. Corticosteroids alone rarely achieve sustained remission and are associated with relapse during tapering. The addition of IVIG within three months of diagnosis markedly improves both biochemical and functional outcomes, particularly in anti-HMGCR disease, where remission rates reach 70-80% [50,57,73-75]. In anti-SRP myopathy, outcomes improve with B-cell depletion using rituximab, which yields clinical improvement in approximately 65-70% of refractory patients [51,75,78]. Steroid-sparing agents such as mycophenolate, azathioprine, or methotrexate maintain remission and reduce relapse below 20% when continued for at least one year [48,73,76-82]. Calcineurin inhibitors (tacrolimus, cyclosporine) show particular benefit in Asian anti-SRP cohorts, achieving partial or complete response in ~60% [48,79-80]. Adjunctive therapies targeting humoral pathways are emerging. FcRn antagonists (efgartigimod, rozanolixizumab) accelerate IgG catabolism and have produced rapid CK reductions in early case reports, while complement inhibitors (eculizumab, zilucoplan) disrupt terminal pathway activation and show encouraging responses in refractory disease [9,37,81,82]. These biologic agents illustrate a shift toward precision therapy aimed at antibody- and complement-driven injury rather than nonspecific immunosuppression.

Prognosis and Relapse Dynamics

Despite high initial response rates, relapse remains a significant challenge, occurring in 10-20% of patients-typically during rapid steroid taper or following premature cessation of immunosuppression [64,66,79]. Predictors include persistent antibody positivity, delayed IVIG initiation, and early steroid withdrawal [87,88]. Maintenance therapy with mycophenolate or azathioprine effectively suppresses subclinical immune reactivation and prevents recurrence by sustaining B-cell quiescence [80,81]. The absence of early IVIG and the presence of cardiac involvement are the strongest negative prognostic indicators [27,52,85].

Safety Considerations

Overall, treatments are well tolerated. IVIG infusions cause mild headaches or transient hypertension in <10% of patients, while serious complications such as thrombosis or aseptic meningitis are rare (<2%) [61,63]. Rituximab is associated with mild upper-respiratory infections in 5-10% and serious infections in only 2-4%, mitigated by vaccination and hepatitis-B prophylaxis [68-70,72]. Cyclophosphamide use is limited by cytopenia and hemorrhagic cystitis [83,84]. No treatment-related deaths were reported, and glucocorticoid-induced metabolic effects remain the main long-term toxicity. Table 4 provides a side-by-side comparison of demographics, triggers, CK peaks, histology, treatment responsiveness, relapse risk, cardiac involvement, and overall outcomes for anti-HMGCR versus anti-SRP IMNM.

**Table 4:** Comparative clinical, laboratory, and treatment outcomes in anti-HMGCR and anti-SRP IMNM Author-Generated Synthesis; no reproduced material. Abbreviations: Anti-HMGCR: anti-3-hydroxy-3-methylglutaryl-coenzyme A reductase; Anti-SRP IMNM: Anti-signal recognition protein Immune-mediated necrotizing myopathies; CK: Creatine kinase; ULN: Upper limit of normal

Domain	Anti-HMGCR	Anti-SRP	Representative Refs
Typical age / sex	45–70 yrs; female > male	20–45 yrs; no sex bias	[21, 27, 43]
Trigger	Statin exposure 60–80%	De novo onset	[7, 24]
CK peak	10–20× ULN	> 20× ULN	[21, 45]
Histology	Necrosis + regeneration	Necrosis > regeneration	[44, 55]
IVIG response	70–80%	≈ 50%	[50, 73]
Relapse rate	20–30%	40–50%	[27, 45]
Cardiac involvement	≈ 5%	10–20%	[52, 67]
Overall outcome	Good recovery	Residual weakness	[45, 49, 86]

Emerging Directions

Novel immunomodulators and complement-pathway inhibitors promise to expand treatment options for refractory disease. Ongoing clinical trials of FcRn antagonists and C5 blockers will clarify their role in sustained antibody suppression. Future work should also address the mechanisms underlying relapse and the potential utility of antibody titer monitoring and MRI as surrogate biomarkers of remission. Early, aggressive combination therapy tailored to serologic subtype offers the best chance of durable recovery and functional restoration.

Quality of Evidence

In summary, this systematic review synthesizes contemporary evidence on antibody-specific mechanisms, clinical outcomes, and therapeutic responses in immune-mediated necrotizing myopathy (IMNM). The findings highlight distinct clinical trajectories between anti-HMGCR and anti-SRP subtypes, emphasizing the central role of early immunosuppression and individualized biologic strategies. Despite heterogeneity in study design, the evidence consistently supports a humoral-complement-macrophage axis of muscle injury and underscores the clinical importance of antibody-guided therapy selection. The overall quality of evidence was moderate, as determined by structured appraisal using JBI, NIH/NOS, ROBINS-I, and AMSTAR-2 instruments. Approximately 82% of included studies met ≥75% of methodological criteria, indicating acceptable internal validity across most datasets. The primary limitations stem from small sample sizes, retrospective design, and incomplete long-term follow-up. Nonetheless, the cumulative body of literature provides a reliable foundation for current clinical practice and future prospective investigations.

Further multicenter, prospective studies are needed to define standardized treatment algorithms, relapse predictors, and biomarkers of therapeutic response across IMNM subtypes. Such studies, ideally incorporating uniform diagnostic criteria and longitudinal functional outcomes, will be essential to advance precision-based management of this rare but increasingly recognized myopathy.

## Conclusions

Immune-mediated necrotizing myopathy (IMNM) represents a distinct, antibody-defined inflammatory myopathy characterized by severe, rapidly progressive weakness and high relapse potential. This systematic review integrates evidence from 88 studies published between 2011 and 2025, demonstrating that disease expression, treatment response, and prognosis differ markedly between anti-HMGCR and anti-SRP subtypes. Early initiation of high-dose corticosteroids combined with IVIG or steroid-sparing immunosuppressants remains the cornerstone of therapy, while emerging biologic agents targeting complement and FcRn pathways show promising results in refractory cases. The overall quality of evidence was moderate, supporting the reliability of current conclusions but emphasizing the need for standardized diagnostic criteria, prospective multicenter trials, and long-term outcome studies. Continued investigation into antibody-specific mechanisms and biomarkers of treatment response will be crucial for developing precision-based therapeutic strategies in IMNM.

## Appendices

**Table 5:** Summary of methodological quality and risk-of-bias appraisal across 88 included studies on immune-mediated necrotizing myopathy (IMNM). Appraisal tools were selected according to study design. Scores represent the proportion of criteria fulfilled per checklist, expressed as mean ± standard deviation. Overall mean quality score: 82% (range 70–95%) Interpretation: Most included studies demonstrated acceptable methodological quality, supporting the reliability of the synthesized evidence.

Study Type	Quality Appraisal Tool	Key Domains Assessed	Studies (n)	Criteria Met (% ± SD)	Overall Rating	Common Limitations
Case reports / Case series	Joanna Briggs Institute (JBI) Checklist	Diagnostic clarity, intervention description, outcome reporting, adequacy of follow-up	45	78 ± 12%	Moderate quality	Small sample size, incomplete follow-up, limited generalizability
Observational cohort / cross-sectional studies	NIH Quality Assessment Tool / Newcastle–Ottawa Scale (NOS)	Population definition, exposure/outcome measurement, control for confounding, attrition bias	25	83 ± 9%	Moderate–High quality	Retrospective design, lack of control group, incomplete longitudinal data
Non-randomized interventional studies	ROBINS-I (Risk Of Bias In Non-Randomized Studies – of Interventions)	Confounding, intervention classification, deviations from intended interventions, missing data, selective reporting	8	80 ± 10%	Moderate quality	Heterogeneous interventions, incomplete blinding
Systematic reviews / meta-analyses (secondary sources)	AMSTAR-2 (A MeaSurement Tool to Assess Systematic Reviews)	Research question clarity, search adequacy, synthesis methods, bias in included data	5	85 ± 8%	High quality	Incomplete reporting of primary-study bias domains

**Table 6:** Appendix A. Citation-Mapping Table (References 1–88) — summarizes section-level citation use

Ref #	First author (year)	Section used	Key sentence / concept supported
1	Mammen AL (2016)	Intro, Background	Defines statin-associated autoimmune myopathy as prototype IMNM; high CK, proximal weakness.
2	Tanaka M (2016)	Treatment	Practical management of statin-triggered IMNM in Japanese patients.
3	Oh EK (2023)	Results, Imaging	Korean anti-HMGCR cohort; clinical + radiologic features.
4	Allenbach Y (2018, Neurology)	Pathogenesis, Discussion	Necrosis in anti-SRP/anti-HMGCR via autoantibodies and complement.
5	Mohassel P, Mammen AL (2013)	Serology	Anti-HMGCR antibodies identified; link to statins.
6	Khoo T, Chinoy H (2023)	Discussion	Unresolved issues and long-term follow-up in anti-HMGCR IMNM.
7	Ma X, Bu BT (2022)	Background	Current concepts in anti-SRP IMNM.
8	Musset L et al. (2016)	Methods, Serology	Multicenter validation of anti-HMGCR testing/ELISA.
9	Martínez-Rodríguez P (2024)	Treatment (rescue)	Plasma exchange in anti-SRP myopathy — partial responses.
10	Sener S (2023)	Results, Pediatric	Refractory relapsing anti-HMGCR in a child.
11	Aussy A (2017)	Discussion	IMNM/dermatomyositis as a window on autoimmunity and cancer.
12	Turner RM (2019)	Background	Statin-related myotoxicity, pharmacogenomics.
13	Yerolatsite M (2025)	Novel therapy	PCSK9 inhibitors safely used after statin-associated IMNM.
14	Damoiseaux J (2019)	Serology	Autoantibodies in IIM; lab evaluation relevant to IMNM.
15	Wang CH (2023)	Epidemiology, Pediatric	Pediatric IMNM features and treatment.
16	Anquetil C (2019)	Immunology	Myositis-specific antibodies as diagnostic cornerstone in IMNM.
17	Xiong A (2021)	Treatment	Rituximab in IMNM — review of case reports/series.
18	Liu M (2023)	Results	Cardiac involvement and its prognostic impact in IMNM.
19	Zhen C (2022)	Treatment	Systematic review of rituximab in inflammatory myopathies (incl. IMNM).
20	Allenbach Y (2020, Nat Rev Rheumatol)	Discussion, Overview	Comprehensive review of IMNM spectrum and subtypes.
21	Dallevet CA, Benveniste O, Allenbach Y (2023)	Discussion	Pathogenesis and treatment update in IMNM.
22	Lundberg IE (2017)	Methods	International myositis classification — supports inclusion criteria.
23	Skolka MP (2025)	Results	Emerging atypical/overlap IMNM manifestations.
24	Yamashita S (2025)	Diagnostics	Autoantibodies as diagnostic keys and disease drivers.
25	Watanabe Y (2024)	Results	Anti-SRP antibodies with myocarditis in overlap setting.
26	Mammen AL (2011)	Intro	Original anti-HMGCR autoantibodies in statin-associated autoimmune myopathy.
27	Allenbach Y (2016)	Results, Prognosis	Anti-SRP has worse prognosis and higher relapse than anti-HMGCR.
28	Arouche-Delaperche / (anti-HMGCR complement) 2017*	Pathogenesis	Anti-HMGCR antibodies induce complement-mediated cytotoxicity.
29	Mescam-Mancini L (2012)	Results	Clinical spectrum and outcomes of anti-SRP necrotizing myopathy.
30	Musset L (2016, ELISA)	Methods	ELISA detection of anti-HMGCR autoantibodies.
31	Jaskowski TD (2017)	Methods	Lab protocol for anti-HMGCR ELISA detection.
32	Nelke C (2024)	Pathology	Complement and MHC patterns provide diagnostic framework.
33	Fionda L (2023)	Imaging	Muscle MRI patterns in IMNM (n = 22); edema → fatty replacement.
34	Dalakas MC (2022)	Discussion, Treatment rationale	Complement in autoimmune inflammatory myopathies; therapeutic implications.
35	Honda M (2024)	Pathology	Microangiopathy, complement, and inflammation in IIM/IMNM muscle.
36	Martins EF (2025)	Background	Pathogenesis linked to clinical features in IIMs incl. IMNM.
37	Peng Q (2025)	Emerging therapy	Efgartigimod (FcRn blockade) as rescue therapy in refractory IMNM.
38	Hanaoka H (2016)	Epidemiology	Anti-SRP antibodies seen even without classic inflammatory myopathy.
39	Engel AG (1991)	Pathology	Early complement MAC deposits in necrotizing myopathies.
40	Werner JL (2012)	Serology	Anti-HMGCR antibody levels correlate with CK and strength.
41	Christopher-Stine L (2010)	Intro	Novel 200/100-kD autoantibodies in necrotizing myopathy.
42	Ge Y (2015)	Epidemiology	Chinese anti-HMGCR cohort; statin exposure data.
43	Alshehri A (2015)	Results	Clinical phenotype of anti-HMGCR myopathy.
44	Suzuki S, Uruha A (2017)	Histology	Autoantibody–pathology association in inflammatory myopathies.
45	Chen Y, Zhu L (2025)	Prognosis	Long-term outcomes of IIM; relapse and recovery patterns.
46	Fang D (2024)	Case-based	Severe anti-SRP IMNM with ARDS — demonstrates refractory potential.
47	Shimoyama T (2024)	Imaging	MRI patterns correlating with clinical features in IIM/IMNM.
48	Ge Y, Zhou H (2015)	Treatment	Tacrolimus in refractory inflammatory myopathies (supports SRP arm).
49	Yoon J (2025)	Clinical management	Statin-associated anti-HMGCR IMNM cohort and outcomes.
50	Mohassel P, Landon-Cardinal O, Mammen AL (2018)	Treatment	Anti-HMGCR myopathy clinical course; IVIG 70–80% response.
51	Kassardjian CD (2015)	Outcomes	Clinical features and treatment outcomes of necrotizing autoimmune myopathy.
52	Pinal-Fernández I (2018)	Background	IMNM overview — demographics, organ involvement.
53	Culvenor AG (2017)	Cardiac	Cardiac involvement in IMNM.
54	Werner JL, Christopher-Stine L (2020)	Practice	Practical management of statin-triggered IMNM.
55	Dalakas MC (2008)	Mechanism	Mechanisms of IVIG in autoimmune neuromuscular disease.
56	Allenbach Y (2020, Brain)	Outcomes	Autoantibodies and outcomes in IMNM.
57	Mammen AL (2015)	Treatment	IVIG monotherapy for statin-triggered autoimmune myopathy.
58	Khoo T (pop. incidence) (2025)	Epidemiology	Population incidence of anti-HMGCR IMNM.
59	Yang MT (2025)	Pediatric vs adult	Imaging and pathology differences in pediatric vs adult anti-HMGCR.
60	Day JA, Limaye V (2019)	Review	IMNM: critical review of current concepts.
61	Kusumoto T (2018)	Overlap / ILD	Necrotizing myopathy after ILD with anti-SRP.
62	Benveniste O, Stenzel W (2016)	Diagnostics	Advances in serological diagnostics of inflammatory myopathies.
63	Rime D. et al. (2024)	Diagnostics	Updated serologic testing for inflammatory myopathies.
64	Álvarez-Troncoso J, Milisenda JC (2025)	Treatment response	Autoantibody profiles and treatment response in IIM.
65	Wang JX (2022)	Outcomes	Outcome predictors in IMNM — early IVIG, lower CK.
66	Portela-Sánchez S (2025)	Epidemiology	IMNM as an emerging disorder (real-world series).
67	Alexandru C (2024)	Cardiac/overlap	Anti-SRP antibodies and myocarditis in IMNM overlap.
68	Szczesny P (2022)	Serology	Screening for anti-HMGCR — variable statin exposure.
69	Ramanathan S (2015)	Clinical course	Anti-HMGCR antibody-associated NAM — treatment and course.
70	Julien S (2024)	Review	“IMNM: a story of antibodies” — narrative overview.
71	Salajegheh M, Amato AA (2025)	Background	Idiopathic inflammatory myopathies — positioning IMNM.
72	Picard C (2016)	Diagnostics	Anti-SRP myositis spectrum; importance of detection method.
73	Suh J, Amato AA (2024)	Management	Management of IMNM — algorithm cited in treatment section.
74	Tiniakou E (2017)	Prognosis	Younger anti-HMGCR patients have more severe disease, slower recovery.
75	Landon-Cardinal O (2019)	Treatment (rescue)	Rituximab in refractory anti-HMGCR IMNM — ≈70% response.
76	Rademacher JG (2022)	Outcomes	Anti-HMGCR IMNM single-centre cohort + literature comparison.
77	Basharat P, Christopher-Stine L (2015)	Update	IMNM: update on diagnosis and management.
78	Ashton C (2016)	Outcomes	Australian necrotising autoimmune myopathy experience.
79	Vencovský J (2019)	Review	Idiopathic inflammatory myopathies — framework that includes IMNM.
80	Li C (2024)	Pathogenesis	Comprehensive review of IMNM pathogenesis and treatment.
81	Shakoory B (2023)	Methods analogy	Cited for severity/risk-stratification framework.
82	Suzuki S (2022)	Large SRP series	100 anti-SRP pts — outcomes and therapy.
83	Merlonghi G (2022)	Myopathology	IMNM as a myopathological challenge.
84	Quinn C (2015)	Background	Necrotizing myopathies: an update.
85	De Bleecker JL (2015)	Histopathology	ENMC workshop: pathology diagnosis of idiopathic inflammatory myopathies, incl. IMNM.
86	Mengge Yang (2025)	Discussion	Pathogenesis and therapeutic implications in IMNM.
87	Howard JF (2021)	Emerging therapy	Efgartigimod (FcRn blockade) in MG — extrapolated to IMNM FcRn strategies.
88	Tang Q (2024)	Results	Clinical characteristics of anti-SRP IIM — supports higher relapse, younger age.
